# Enhancing Yogurt Functionality: Effects of *Aronia melanocarpa* and *Lactobacillus acidophilus* DSM 20079 on Quality Parameters and In Vitro Bioaccessibility

**DOI:** 10.1002/fsn3.72337

**Published:** 2026-09-24

**Authors:** Çiğdem Konak Göktepe, Neşe Mutlu, Talha Demirci, Aysun Oraç, Nihat Akin, Lütfi Pirlak

**Affiliations:** ^1^ Department of Food Engineering, Faculty of Agriculture Selcuk University Konya Türkiye; ^2^ Department of Food Processing, Karapınar Aydoğanlar Vocational School Selcuk University Konya Türkiye; ^3^ Department of Horticulture, Faculty of Agriculture Selcuk University Konya Türkiye

**Keywords:** ACE inhibition, alpha‐glucosidase inhibition, antioxidant capacity, functional yogurt, phenolic compounds, probiotics

## Abstract

This study evaluated the impact of *Aronia melanocarpa* puree (2% and 6%) and *Lactobacillus* (*L*.) *acidophilus* DSM 20079 on yogurt quality during 28 days of storage, with emphasis on physicochemical, microbiological, and biofunctional properties. Acidification kinetics varied with formulation, with yogurt B (6% aronia+probiotic) reaching pH 4.6 faster (194 ± 2 min) than yogurt A (2% aronia+probiotic; 233 ± 2 min). The addition of aronia reduced hardness while enhancing water holding capacity. It also enhanced functional properties, with 6% aronia and *L. acidophilus* DSM 20079 increasing total phenolic content by 71.04% and DPPH activity by 414.93% compared to the control. Yogurt B exhibited the highest α‐glucosidase inhibitory activity on Day 28 (33.23%), whereas the control showed 12.21%. ACE inhibitory activity was enhanced only in probiotic‐containing yogurts. In addition, antioxidant bioaccessibility peaked during intestinal digestion, where FRAP values increased by up to 435.80%. Sensory evaluation indicated that the addition of probiotics to aronia‐enriched yogurts positively influenced overall sensory quality scores, although stirred‐type samples received the lowest scores (5.08) by Day 28. Throughout storage, *L. acidophilus* DSM 20079 counts remained above the recommended probiotic threshold of 6 log CFU/g, ensuring the functional probiotic potential of the product. These findings highlight the potential of aronia and probiotic supplementation to improve the functional and sensory properties of yogurt, offering a promising strategy for the development of functional dairy products.

## Graphical abstract

*Aronia melanocarpa* puree (2% and 6%) was incorporated into yogurt with or without *Lactobacillus acidophilus* DSM 20079. The treatments affected acidification kinetics and textural properties, resulting in reduced hardness and increased water‐holding capacity. Aronia and probiotic supplementation enhanced antioxidant activity, total phenolic content, and α‐glucosidase inhibition. ACE inhibitory activity was observed in aronia yogurt containing *L. acidophilus* DSM 20079, while probiotic supplementation improved the sensory acceptability of aronia yogurt.

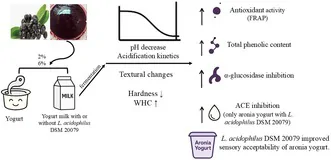

## 1 Introduction

In the functional food market, fruit‐enriched probiotic yogurts stand out as products that offer both rich phenolic content and support for the viability of probiotics (Dimitrellou et al. 2020). Yogurt is considered an ideal carrier for the stable delivery of probiotic microorganisms due to its low pH, high protein, and moisture content, while the addition of fruit contributes to the formulation by enhancing both functional value and sensory diversity (Meybodi et al. 2020). Probiotics are live microorganisms that, when consumed in adequate amounts (≥ 10^6^ CFU/g), can confer various health benefits to the host (Terpou et al. 2019). Although their potential effects depend on several factors such as strain characteristics, dosage, carrier matrix, and individual physiology, one of the fundamental criteria is their ability to remain viable until the point of consumption (Markowiak and Śliżewska 2017). Starter culture bacteria used in yogurt production generally cannot survive gastrointestinal conditions or establish persistent colonization in the intestine, which limits their potential health benefits (Fazilah et al. 2018). Therefore, probiotic microorganisms capable of withstanding the digestive tract and promoting host health are added to yogurt to enhance its functional properties (Guimarães et al. 2020). Recent studies enhance yogurt by combining probiotics with prebiotics or encapsulation to improve microbial viability and functional properties (Dimitrellou et al. 2025; Laina et al. 2025).

Aronia ( *Aronia melanocarpa* ) is a potent functional food, rich in anthocyanins, flavonoids, and phenolics, providing antioxidant, antidiabetic, anti‐inflammatory, and cardioprotective effects (Ren et al. 2022; Sidor and Gramza‐Michałowska 2019). Although fresh and unprocessed aronia fruits are rarely consumed directly due to their astringent taste, they are widely used in the food industry in the production of a variety of products, ranging from juice to dietary supplements (Bratosin et al. 2024; Du and Myracle 2018a; Yang et al. 2025). Its incorporation into dairy matrices such as yogurt, kefir, and milk has been shown to significantly improve antioxidant capacity, phenolic content, and in some cases, probiotic viability (Kim et al. 2019; Plessas et al. 2023).

While previous studies have demonstrated the functional benefits of aronia in dairy products, no comprehensive study has systematically investigated the combined effects of aronia puree and *L. acidophilus* in yogurt during long‐term storage. In particular, the effects on physicochemical and textural properties, α‐glucosidase and ACE inhibitory activities, in vitro digestibility, and sensory acceptability have not been evaluated together. To address this gap, the present study investigates the effects of aronia puree (2% and 6%) and *L. acidophilus* DSM 20079 supplementation on the physicochemical, microbiological, functional, and sensory properties of set‐ and stirred‐type yogurt over 28 days of refrigerated storage. Additionally, functional parameters such as α‐glucosidase and ACE inhibitory activities, as well as in vitro digestibility and phenolic bioaccessibility, were comprehensively evaluated. This work aims to contribute to the optimization of polyphenol–probiotic combinations in fermented dairy systems, thereby supporting the development of novel functional foods with enhanced health benefits and consumer acceptability.

## 2 Materials and Methods

### 2.1 Materials

Raw cow's milk (pH 6.64; dry matter 14.17%; fat 4.30%; solids‐not‐fat 9.97%) was standardized to 12% skim milk solids with skim milk powder (Enka Süt A.Ş., Türkiye; dry matter 95.48%). Yogurt was prepared using a commercial starter ( *L. delbrueckii* subsp. *bulgaricus* and *S. thermophilus* ), with *L. acidophilus* DSM 20079 as the probiotic strain. Fresh aronia fruits were sourced from experimental fields at Selçuk University (Konya, Türkiye).

### 2.2 Preparation of Aronia Puree

**FIGURE 1:**
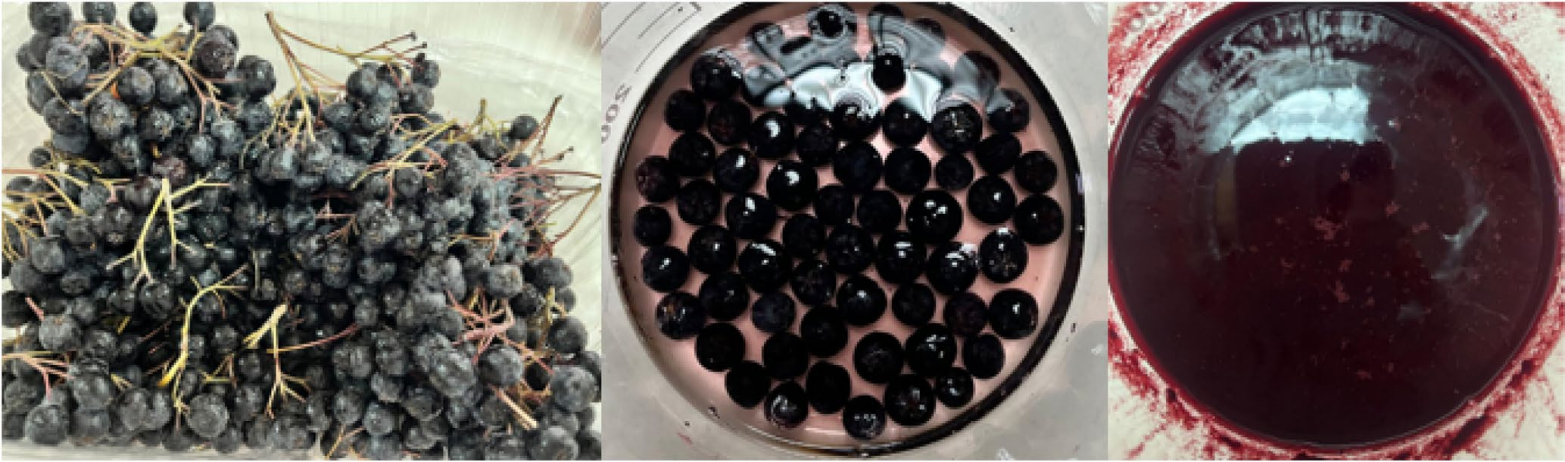
Photographs of aronia fruit and aronia puree.

Fresh aronia fruits (pH 3.63; dry matter 26.16%; color values L* 9.59, a* 16.63, b* −3.47), characterized as dark‐purple mature berries, were blended with water (1:1, w/v), homogenized (Ultra‐Turrax), and pasteurized at 80°C for 10 min. The puree was incorporated into yogurt milk at ~70°C, or at 20°C for stirred‐type yogurt. Representative images are shown in Figure 1.

### 2.3 Production of Yogurt Samples

**FIGURE 2:**
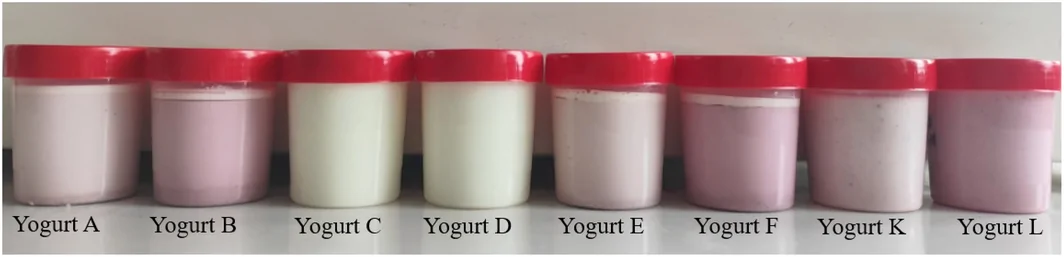
The photograph of yogurt samples. Yogurt (A) set‐type yogurt formulated with 2% aronia puree and *L. acidophilus* DSM 20079; Yogurt (B) set‐type yogurt formulated with 6% aronia puree and *L. acidophilus* DSM 20079; Yogurt (C) set‐type control yogurt formulated with *L. acidophilus* DSM 20079 and without aronia puree; Yogurt (D) set‐type control yogurt formulated without aronia puree and *L. acidophilus* DSM 20079; Yogurt (E) set‐type yogurt formulated with 2% aronia puree and without *L. acidophilus* DSM 20079; Yogurt (F) set‐type yogurt formulated with 6% aronia puree and without *L. acidophilus* DSM 20079; Yogurt (K) stirred‐type yogurt formulated with 2% aronia puree; Yogurt (L) stirred‐type yogurt formulated with 6% aronia puree.

Eight yogurt samples were prepared as set‐ or stirred‐type (Figure 2, Table 1). Raw cow's milk was standardized to 12% non‐fat dry matter, heat‐treated (90°C/10 min), cooled (44°C), and inoculated with 2% (v/v) starter culture ( *L. delbrueckii* subsp. *bulgaricus* and *S. thermophilus* ), with *L. acidophilus* DSM 20079 added at 1% (v/v) for probiotic formulations. For set‐type yogurts, pasteurized milk was cooled to 75°C ± 2°C, supplemented with aronia puree (2% or 6%), homogenized, and inoculated at 44°C, followed by incubation at 42°C to pH 4.6, cooling, and storage at 4°C for 28 days. Stirred‐type yogurts were produced by fermenting milk to pH 4.6, cooling to 20°C, gently breaking the curd, incorporating pasteurized aronia puree (2% or 6%), portioning into 100 g containers, and storing at 4°C for 28 days.

**TABLE 1:** Yogurt sample design according to functional ingredient application and processing method.

Sample code	Manufacturing process	Aronia puree (%)	Probiotic
Yogurt A	Set‐type	2%	+
Yogurt B	Set‐type	6%	+
Yogurt C	Set‐type (control)	−	+
Yogurt D	Set‐type (control)	−	−
Yogurt E	Set‐type	2%	−
Yogurt F	Set‐type	6%	−
Yogurt K	Stirred‐type	2%	−
Yogurt L	Stirred‐type	6%	−

### 2.4 Yogurt Samples Characterization

#### 2.4.1 Fermentation Kinetics and Physicochemical Analyses

Fermentation kinetics were assessed by monitoring the pH decline at 30‐min intervals until the target pH of 4.6 was reached. The acidification rate for each interval was calculated as the change in pH over the change in time (∆pH/∆t). Based on these continuous calculations, three key kinetic parameters were extracted: (1) the maximum acidification rate (*V*_max_), identified as the peak rate of pH decrease during the process and expressed as 10^−3^ upH/min; (2) the time at which this maximum acidification rate occurred (*T*_*V*max_, h); and (3) the total fermentation time required to reach pH 4.6 (*T*_pH4.6_, h) (Gölbaşι et al. 2023). Dry matter (AOAC 990.19), fat (AOAC 989.05), and ash (AOAC 945.46) contents were determined according to AOAC (2005). pH was measured using a calibrated pH meter (WTW 315i Set), and titratable acidity was expressed as % lactic acid after titration with 0.1 N NaOH (AOAC 2005). Color parameters (L*, a*, b*) were recorded using a Minolta CR‐400 colorimeter (McLellan et al. 1995). Water holding capacity (WHC) was assessed by centrifuging 5 g of yogurt at 4500 rpm for 30 min at 10°C, and syneresis was calculated as the percentage of whey drained after 4 h of filtration at 4°C (Gölbaşι et al. 2023).

#### 2.4.2 Hardness Analysis

The hardness of yogurt samples was determined using a TA‐XT2 Texture Analyzer (Stable Micro Systems, Godalming, UK) equipped with a 50 kg load cell. Texture measurements were performed using a penetration test based on the method of Akshit et al. (2025), with appropriate modifications. Before testing, samples were taken from refrigerated storage (4°C ± 1°C) and allowed to equilibrate to room temperature. Penetration measurements were performed on 50 g yogurt portions placed in 100 mL plastic cups using a 36 mm cylindrical probe. The operating conditions were set at a pre‐test speed of 3 mm/s, a test speed of 3.3 mm/s, a post‐compression speed of 3 mm/s, and a penetration depth of 15 mm. Hardness (N), defined as the maximum force required during probe penetration, was recorded for each sample. All measurements were carried out in triplicate on storage Days 1, 14, and 28.

#### 2.4.3 Microbiological Analyses

Selective enumeration of *L. acidophilus* DSM 20079 was performed on MRS agar (Merck, Darmstadt, Germany) supplemented with 10 mg/L bile and clindamycin and incubated anaerobically at 37°C for 48 h (Gebara et al. 2015). *L. delbrueckii* subsp. *bulgaricus* was enumerated on MRS agar (pH 5.2) after anaerobic incubation at 43°C for 72 h, while *S. thermophilus* was cultured on M17 agar (Merck, Darmstadt, Germany) (pH 7.2 ± 0.2) under aerobic conditions at 37°C for 24 h (Tharmaraj and Shah 2003). Yeast and mold counts were determined on YGC agar (Merck, Darmstadt, Germany) after incubation at 25°C under aerobic conditions for 5 days (Varga et al. 2014). All microbiological analyses were conducted in duplicate on Days 1, 14, and 28 of storage, and results were expressed as log CFU/g.

#### 2.4.4 α‐Glucosidase Inhibition Activity

Water‐soluble extracts of the yogurt samples were prepared as described by Shori and Baba (2023). Aqueous extracts were prepared by homogenizing 10 g of yogurt with 2.5 mL of sterile distilled water using an Ultra‐Turrax T25 D (IKA, Germany). The pH was adjusted to 4.0 with 0.1 M HCl, followed by incubation at 45°C for 10 min and centrifugation at 5000 × *g* for 10 min at 4°C. The supernatant was neutralized to pH 7.0 with 0.5 M NaOH, recentrifuged at 9000 rpm for 10 min to achieve clarity and filtered through a 0.45 μm membrane. The resulting aqueous extracts were used for chemical analyses, while excess supernatants were stored at −20°C.

Subsequently, 500 μL of the extract was combined with 1000 μL of α‐glucosidase solution (1.0 U/mL in 0.1 M potassium phosphate buffer, pH 6.90) and incubated at 37°C for 10 min. Subsequently, 500 μL of 5 mM p‐nitrophenyl‐α‐D‐glucopyranoside (pNPG) prepared in the same buffer was added, followed by a further 5 min incubation at 37°C. Absorbance was recorded at 405 nm before and after incubation. The control contained buffer instead of extract.

(Shori and Baba 2023). The α‐glucosidase inhibition activity of yogurts was determined on the 28th day of storage and expressed as percentage inhibition. The percentage of α‐glucosidase inhibition was calculated using the formula:

$$\text{Inhibition}\%=\left[\left({\text{Control}}_{\text{absorbance}}-{\text{Sample}}_{\text{absorbance}}\right)/{\text{Control}}_{\text{absorbance}}\right]\times 100$$

#### 2.4.5 Angiotensin‐Converting Enzyme (ACE) Inhibitor Activity

Water‐soluble extracts of yogurt samples were obtained by centrifuging 40 g of yogurt at 9000 rpm for 10 min at 5°C, followed by filtration through a 0.45 μm membrane, as described by Erkaya‐Kotan (2020). ACE inhibitory activity was measured according to Kariyawasam et al. (2021) by incubating 20 μL of the extract with 90 μL of 5 mM HHL in sodium borate buffer (pH 8.3) at 37°C for 5 min, followed by the addition of 20 μL of ACE enzyme (0.1 U/mL). After incubation for 30 min at 37°C, the reaction was terminated with 0.1 M HCl, and hippuric acid was extracted using ethyl acetate. Absorbance was measured at 228 nm, and ACE inhibition (%) was calculated using the following equation:

$$\begin{array}{c} \mathrm{ACE}\;\text{inhibitory activity}\;\left(\%\right)\\ \;=\left[\left({\text{Control}}_{\text{absorbance}}-{\text{Sample}}_{\text{absorbance}}\right)/{\text{Control}}_{\text{absorbance}}\right]\times 100 \end{array}$$

where Control_absorbance_ and Sample_absorbance_ represent the absorbance values of the control (distilled water) and the aqueous extract of the yogurt samples, respectively.

#### 2.4.6 Total Phenolic Content (TPC), Antioxidant Activity, and In Vitro Digestibility Analyses

Yogurt extracts were prepared by homogenizing 5 g of sample with 25 mL of 80% methanol, followed by centrifugation at 7200 rpm for 10 min at 4°C, filtration (Whatman No. 1), and passage through a 0.45 μm membrane (Demirci et al. 2017). The resulting extracts were used for DPPH and FRAP antioxidant activity assays, as well as TPC determination.

The antioxidant capacity of the samples was determined using the DPPH radical scavenging method according to Brand‐Williams et al. (1995). For the analysis, 100 μL of extract was mixed with 900 μL of Tris–HCl (pH 7.4) and 2 mL of DPPH solution (0.1 mmol/L in methanol), and the mixture was incubated in the dark at room temperature for 30 min. For the control, 80% methanol was used instead of the sample, and absorbance was measured at the 30th minute at 517 nm using a spectrophotometer (Optizen 3220, Korea), with methanol used for baseline correction. The percentage of DPPH inhibition and the TEAC value were calculated using the following equations, and the results were expressed as μM TEAC/mg.

$$\%\text{Inhibition}=\left[\left({\text{Control}}_{\text{absorbance}}-{\text{Sample}}_{\text{absorbance}}\right)/{\text{Control}}_{\text{absorbance}}\right]\times 100$$

$$\text{TEAC}\;\left(\mathrm{\mu M}\;\text{Trolox}/g\;\text{sample}\right)=\left(\%\text{Inhibition}+0.9482\right)/14.084$$

The FRAP analysis was performed according to the method described by Lina Yang et al. (2016). For this assay, 75 μL of sample extract was mixed with 2.25 mL of FRAP reagent and 225 μL of distilled water. The mixture was incubated in the dark at room temperature (25°C ± 1°C) for 30 min, and the absorbance was measured at 593 nm using the same spectrophotometer. Trolox was used to calibrate the standard curve, and the results were expressed as mg Trolox/100 g.

The TPC was determined using the Folin–Ciocalteu method as described by Konić‐Ristić et al. (2011). Briefly, 200 μL of extract was mixed with 1 mL of Folin–Ciocalteu reagent (1:10, v/v), followed by the addition of 800 μL sodium carbonate solution (75 g/L) after 4 min. The mixture was incubated at room temperature for 2 h, and absorbance was then measured at 765 nm. Results were expressed as mg gallic acid equivalents (GAE)/100 g.

Simulated gastrointestinal digestion of yogurts was performed on the 28th day of storage using the standardized INFOGEST 2.0 protocol (Brodkorb et al. 2019). Electrolyte stock solutions (KCl, KH_2_PO_4_, NaHCO_3_, NaCl, MgCl_2_·6H_2_O, and (NH_4_)_2_CO_3_) were prepared in distilled water, and simulated salivary (SSF), gastric (SGF), and intestinal fluids (SIF) were formulated accordingly. Calcium chloride (3.33 g/100 mL) and a 10 mM bile salt solution (0.4086 g/100 mL) were also prepared. For oral digestion, 7 g yogurt was mixed with SSF (final volume 7 mL) and CaCl_2_ (0.035 mL) and incubated at 37°C for 2 min after pH adjustment to 7. In the gastric phase, the oral digest (12 mL) was combined with SGF (final volume 12 mL), CaCl_2_ (6 μL), pepsin (2000 U/mL), and lipase (60 U/mL), adjusted to pH 3, and incubated at 37°C for 2 h. For the intestinal phase, pancreatin (100 U/mL), bile salts (2.5 mL), SIF (11 mL), and CaCl_2_ (40 μL) were added to 20 mL gastric digest, the pH was adjusted to 7, and the final volume was brought to 40 mL with SIF before incubation at 37°C for 2 h. After gastric and intestinal digestion, 1 mL aliquots were mixed with 5 mL of 80% methanol, centrifuged at 7200 rpm for 10 min, and filtered (0.45 μm). The resulting supernatants were used for TPC, DPPH, and FRAP analyses.

#### 2.4.7 Cytotoxicity Assay

The cytotoxic effects of yogurt samples were evaluated using the human colorectal adenocarcinoma cell line (CaCo‐2). Cells were maintained at 37°C in a humidified atmosphere with 5% CO_2_ in DMEM high glucose medium supplemented with 10% FBS, 1% penicillin/streptomycin, and 1% L‐glutamine. Cell viability was assessed by Trypan Blue exclusion after detachment with trypsin/EDTA. For the assay, cells were seeded in 96‐well plates at a density of 3 × 10^3^ cells/well in duplicate. Yogurt extracts were applied at 3% and 5% concentrations in a final volume of 100 μL per well. Negative controls received only culture medium, while positive controls were treated with 10% DMSO. Solvent controls were treated with ultrapure distilled water (UP dH_2_O) at equivalent concentrations. Following 24 and 48 h of incubation, media were removed and replaced with fresh medium containing 10% CCK‐8 solution. Cells were incubated at 37°C in the dark for up to 3 h. Cell viability was measured at 450 nm using an Epoch 2 microplate spectrophotometer (BioTek, Winooski, VT, USA). Viability ratios were calculated relative to the control group (Zhao et al. 2013).

#### 2.4.8 Sensory Analysis

Sensory profiling and quality evaluation of yogurt samples were performed according to the descriptive quality scoring principles described by Lawless and Heymann (2010), following the yogurt sensory evaluation protocol reported by Denktaş et al. (2025). Yogurt samples were evaluated on the 1st, 14th, and 28th day of refrigerated storage by a panel of nine trained panelists, all of whom were academicians with expertise in the sensory evaluation of yogurt. Appearance, aroma, mouthfeel, taste, texture, and overall quality were assessed on a nine‐point quality scoring scale (1 = extremely poor/unacceptable, 9 = excellent). To prevent any bias and ensure blinding of the panelists, the yogurt samples were coded with randomized codes to prevent any bias. All panelists evaluated the samples independently under controlled conditions.

### 2.5 Statistical Analysis

All analytical procedures were conducted in triplicate (*n* = 3), except for microbiological analyses, which were performed in duplicate. Samples were randomly assigned to positions during fermentation and refrigerated storage, and all analyses were conducted in a randomized order. Statistical analyses were performed using Minitab Statistical Software (Version 22, Minitab LLC, State College, PA, USA). Data were analyzed by one‐way ANOVA followed by Tukey's post hoc test, with statistical significance set at *p* < 0.05.

## 3 Results and Discussion

### 3.1 Fermentation Kinetics and Physicochemical Characteristics of Yogurts

**FIGURE 3:**
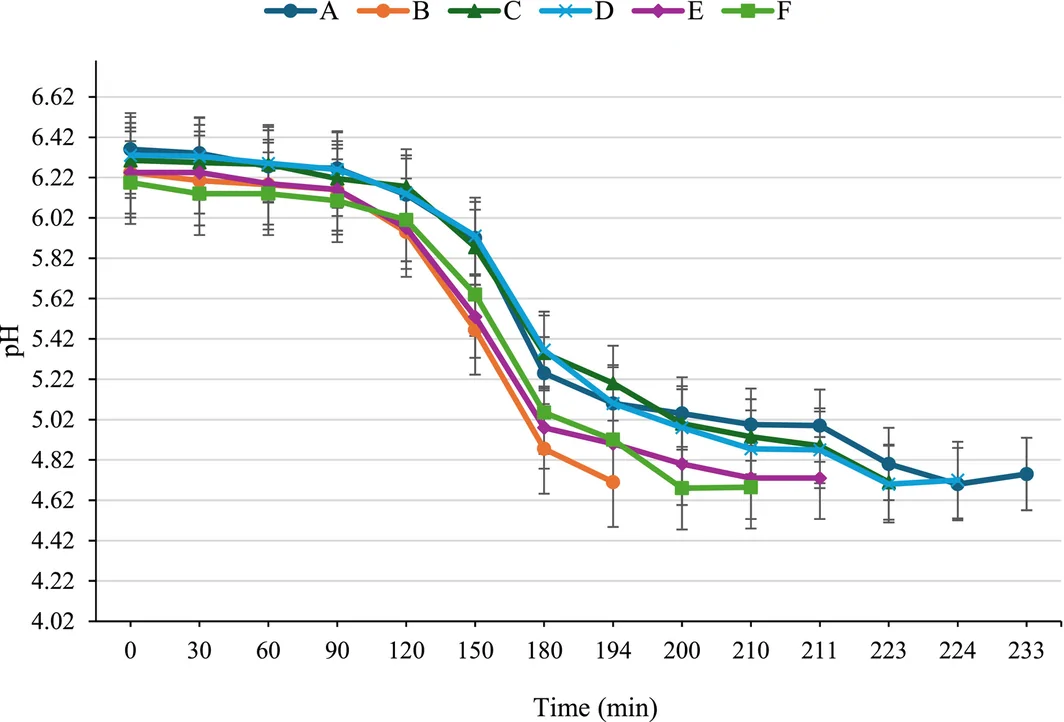
Fermentation dynamics of yogurt samples during incubation. Yogurt A, set‐type yogurt formulated with 2% aronia puree and *L. acidophilus* DSM 20079; Yogurt B, set‐type yogurt formulated with 6% aronia puree and *L. acidophilus* DSM 20079; Yogurt C, set‐type control yogurt formulated with *L. acidophilus* DSM 20079 and without aronia puree; Yogurt D, set‐type control yogurt formulated without aronia puree and *L. acidophilus* DSM 20079; Yogurt E, set‐type yogurt formulated with 2% aronia puree and without *L. acidophilus* DSM 20079; Yogurt F, set‐type yogurt formulated with 6% aronia puree and without *L. acidophilus* DSM 20079.

Acidification kinetics of yogurts during fermentation are presented in Figure 3. The pH values remained almost constant during the initial 90 min, representing the microbial adaptation phase (Bezerra et al. 2012). After 120 min of fermentation, a rapid decrease in pH was observed, with yogurt B (6% aronia + probiotics) exhibiting the fastest acidification. It should be noted that the formulations containing 6% aronia puree exhibited slightly lower initial pH values at the beginning of fermentation. Specifically, yogurt B (6.245) and yogurt F (6.195) showed lower initial pH values than the control yogurts C (6.305) and D (6.330). Since the aronia puree itself had a low pH (3.63), this difference may be associated with its incorporation into the yogurt formulation. Therefore, the faster pH decline observed in yogurt B may reflect both its lower initial pH and microbial acidification during fermentation. While yogurt A (2% aronia + probiotics) required the longest time (233 min) to reach pH 4.6, among the control groups, sample D (without probiotics) completed fermentation 24 min later than sample C (with probiotics). Moreover, the acidification kinetics results presented in Table 2 indicate that the Vmax values of the samples were comparable, with no statistically significant differences observed between the groups (*p* > 0.05). The combination of 2% aronia puree and probiotic culture (yogurt A) prolonged the *T*_*V*max_, while the addition of 6% aronia (yogurt B) reduced this time. Consistent with our findings, Sanusi et al. (2022) reported that incorporating graviola puree reduced the *T*_*V*max_ value of yogurt samples suitable for incubation. The fermentation time (*T*_pH4.6_) in all yogurts varied within a narrow range, and no significant differences were observed (*p* > 0.05).

**TABLE 2:** The acidification kinetics parameters and some chemical properties of the yogurts.

Yogurts^†^	*T*_*V*max_ (h)	*V*_max_ (10^−3^ upH/min)	*T*_pH 4.6_ (h)	Dry matter (%)	Fat (%)	Ash (%)
A	3.00 ± 0.00^A^	22.33 ± 1.41^A^	3.89 ± 0.03^A^	14.27 ± 0.18^C^	4.02 ± 0.03^AB^	0.96 ± 0.02^CB^
B	2.75 ± 0.35^A^	19.83 ± 1.18^A^	3.23 ± 0.04^A^	14.12 ± 0.44^C^	3.95 ± 0.07^B^	0.94 ± 0.00^C^
C	3.25 ± 0.35^A^	25.00 ± 0.94^A^	3.71 ± 0.11^A^	16.00 ± 0.18^A^	4.15 ± 0.07^AB^	1.05 ± 0.06^AB^
D	3.25 ± 0.35^A^	21.17 ± 1.65^A^	3.73 ± 0.07^A^	15.48 ± 0.09^AB^	4.17 ± 0.03^A^	1.07 ± 0.01^AB^
E	2.75 ± 0.35^A^	22.00 ± 4.71^A^	3.51 ± 0.00^A^	15.28 ± 0.11^B^	4.05 ± 0.07^AB^	1.11 ± 0.02^A^
F	3.00 ± 0.00^A^	19.50 ± 2.12^A^	3.33 ± 0.44^A^	15.22 ± 0.09^B^	4.07 ± 0.03^AB^	0.97 ± 0.00^CB^
K	—	—	—	13.93 ± 0.29^C^	4.15 ± 0.07^AB^	0.96 ± 0.01^CB^
L	—	—	—	14.13 ± 0.03^C^	4.17 ± 0.03^A^	0.93 ± 0.02^C^

*Note:* Different letters within a column indicate significant differences among means (*p* < 0.05).

^†^ Yogurt A, set‐type yogurt formulated with 2% aronia puree and *L. acidophilus* DSM 20079; Yogurt B, set‐type yogurt formulated with 6% aronia puree and *L. acidophilus* DSM 20079; Yogurt C, set‐type control yogurt formulated with *L. acidophilus* DSM 20079 and without aronia puree; Yogurt D, set‐type control yogurt formulated without aronia puree and *L. acidophilus* DSM 20079; Yogurt E, set‐type yogurt formulated with 2% aronia puree and without *L. acidophilus* DSM 20079; Yogurt F, set‐type yogurt formulated with 6% aronia puree and without *L. acidophilus* DSM 20079; Yogurt K, stirred‐type yogurt formulated with 2% aronia puree; Yogurt L, stirred‐type yogurt formulated with 6% aronia puree.

As expected, the addition of aronia puree caused an overall decrease in the dry matter, fat, and ash contents of the yogurts. The reductions in dry matter and ash were statistically significant (*p* < 0.05), whereas the change in fat content was not significant (Table 2). These changes can be attributed to compositional differences between the standardized milk base and the aronia puree. Although fresh aronia contained 26.16% dry matter, the puree was prepared with a 1:1 (w/v) water‐to‐fruit ratio, resulting in lower solids than the milk base. Moreover, the relatively low ash content of *A. melanocarpa* reported in the literature may also have contributed to the reduction in ash content observed in yogurts (Kulling and Rawel 2008). Similarly, Pădureţ et al. (2024) reported no significant effect of aronia supplementation on fat content, consistent with the present findings. However, unlike the present study, they observed a slight increase in dry matter following aronia addition.

Throughout the 28‐day storage period, set‐type yogurts containing both aronia and probiotics (A and B), as well as the probiotic control yogurt (C), exhibited significantly lower pH values compared to their non‐probiotic counterparts, with no statistically significant effect observed for aronia concentration (Table 3). Moreover, post‐acidification was more pronounced in stirred‐type yogurts (K and L) compared to set‐type samples. Notably, titratable acidity peaked on Day 14 in yogurts K and L; however, these differences were no longer statistically significant by Day 28. These results suggest that the addition of aronia and probiotics influenced the acidity profile of yogurts in a time‐dependent manner during fermentation and storage. The observed increase in acidity may be attributed to the metabolic activity of *L. acidophilus* DSM 20079 (Moayednia et al. 2009) and the organic acids naturally present in aronia puree (Gerasimov et al. 2023). Post‐acidification is generally considered to result from the persistence of metabolic activity by starter cultures and probiotic bacteria under refrigerated conditions, despite the reduced fermentation rate (He et al. 2026). The gradual utilization of residual fermentable substrates may contribute to continued organic acid production during storage (Zhang et al. 2024). Similar to our results, Molaee Parvarei et al. (2021) reported that yogurts containing probiotics had higher acidity values during storage due to post‐acidification than yogurts without probiotics.

**TABLE 3:** The pH, titratable acidity, water holding capacity, and syneresis values of yogurts during cold storage.

	Yogurts^†^	Storage period (day)		
		1	14	28
pH	A	4.01 ± 0.00^D,a^	4.05 ± 0.05^B,a^	3.94 ± 0.07^AB,a^
	B	4.13 ± 0.01^BCD,a^	4.03 ± 0.01^bc,b^	3.94 ± 0.02^AB,c^
	C	4.19 ± 0.03^ABC,a^	4.12 ± 0.00^AB,ab^	4.01 ± 0.04^AB,b^
	D	4.31 ± 0.04^A,a^	4.16 ± 0.02^AB,ab^	4.05 ± 0.07^A,b^
	E	4.18 ± 0.00^ABC,a^	4.15 ± 0.00^AB,a^	3.94 ± 0.02^AB,b^
	F	4.21 ± 0.02^AB,a^	4.21 ± 0.04^A,a^	3.95 ± 0.00^AB,b^
	K	4.06 ± 0.07^CD,a^	3.87 ± 0.07^CD,a^	3.83 ± 0.02^B,a^
	L	4.06 ± 0.03^CD,a^	3.78 ± 0.01^D,b^	3.89 ± 0.08^AB,ab^
Titratable acidity (% lactic acid)	A	1.27 ± 0.08^A,b^	1.46 ± 0.02^AB,ab^	1.59 ± 0.04^A,a^
	B	1.16 ± 0.00^AB,b^	1.36 ± 0.00^ABC,a^	1.41 ± 0.03^A,a^
	C	1.16 ± 0.04^AB,b^	1.25 ± 0.05^C,b^	1.49 ± 0.05^A,a^
	D	1.04 ± 0.02^bc,b^	1.23 ± 0.03^C,ab^	1.44 ± 0.09^A,a^
	E	1.18 ± 0.08^AB,b^	1.35 ± 0.03^ABC,b^	1.65 ± 0.08^A,a^
	F	0.93 ± 0.01^C,b^	1.29 ± 0.02^bc,a^	1.52 ± 0.13^A,a^
	K	1.12 ± 0.06^ABC,b^	1.49 ± 0.08^A,a^	1.58 ± 0.01^A,a^
	L	1.26 ± 0.04^A,b^	1.51 ± 0.07^A,a^	1.59 ± 0.05^A,a^
Water holding capacity (%)	A	49.25 ± 2.18^AB,a^	51.77 ± 0.27^AB,a^	50.44 ± 0.91^AB,a^
	B	50.59 ± 0.14^A,a^	53.37 ± 1.42^A,a^	52.70 ± 1.83^A,a^
	C	44.35 ± 0.06^C,a^	46.56 ± 0.21^D,a^	47.85 ± 1.62^AB,a^
	D	45.76 ± 0.92^bc,a^	47.42 ± 0.55^D,a^	47.75 ± 0.35^AB,a^
	E	45.37 ± 2.18^bc,a^	47.77 ± 0.21^CD,a^	46.00 ± 2.54^B,a^
	F	47.90 ± 0.98^ABC,a^	48.80 ± 1.41^BCD,a^	48.75 ± 1.34^AB,a^
	K	52.09 ± 0.42^A,a^	52.83 ± 0.23^A,a^	51.05 ± 1.06^AB,a^
	L	49.55 ± 0.35^AB,a^	51.27 ± 1.52^ABC,a^	49.25 ± 0.35^AB,a^
Syneresis (%)	A	35.07 ± 1.30^CD,a^	32.28 ± 0.25^B,ab^	30.62 ± 0.96^BCD,b^
	B	34.62 ± 1.37^CD,a^	35.07 ± 2.75^AB,a^	29.30 ± 1.47^CD,a^
	C	35.02 ± 0.26^CD,a^	27.55 ± 1.02^C,b^	34.85 ± 1.21^AB,a^
	D	37.49 ± 0.29^bc,a^	34.46 ± 0.46^B,b^	24.65 ± 0.66^E,c^
	E	36.79 ± 0.45^BCD,a^	30.92 ± 0.88^bc,b^	28.12 ± 0.94^DE,b^
	F	38.52 ± 1.18^AB,a^	34.92 ± 0.61^B,a^	28.57 ± 1.48^DE,b^
	K	41.15 ± 0.64^A,a^	33.98 ± 0.24^B,b^	33.61 ± 1.07^ABC,b^
	L	33.97 ± 0.38^D,b^	39.44 ± 0.21^A,a^	35.11 ± 0.63^A,b^

*Note:* Different uppercase letters within the same column indicate significant differences among samples, while different lowercase letters within the same row indicate significant differences during storage (*p* < 0.05).

^†^ Yogurt A, set‐type yogurt formulated with 2% aronia puree and *L. acidophilus* DSM 20079; Yogurt B, set‐type yogurt formulated with 6% aronia puree and *L. acidophilus* DSM 20079; Yogurt C, set‐type control yogurt formulated with *L. acidophilus* DSM 20079 and without aronia puree; Yogurt D, set‐type control yogurt formulated without aronia puree and *L. acidophilus* DSM 20079; Yogurt E, set‐type yogurt formulated with 2% aronia puree and without *L. acidophilus* DSM 20079; Yogurt F, set‐type yogurt formulated with 6% aronia puree and without *L. acidophilus* DSM 20079; Yogurt K, stirred‐type yogurt formulated with 2% aronia puree; Yogurt L, stirred‐type yogurt formulated with 6% aronia puree.

As seen in Table 3, the supplementation of aronia puree and probiotic cultures significantly influenced the WHC and syneresis of the yogurts. Yogurt B (6% aronia + probiotics) exhibited the highest WHC and lowest syneresis on the 28th day of storage, confirming the inversely proportional relationship between these parameters. Similarly, yogurt L (stirred‐type, with 6% aronia) also showed high WHC with reduced syneresis, likely due to the interaction between aronia‐derived polyphenols and pectin with the casein matrix, enhancing water retention and reducing whey separation (Du et al. 2021; Nawirska and Kwaśniewska 2005; Nguyen and Hwang 2016). However, yogurt K (stirred‐type, 2% aronia) demonstrated both high WHC and high syneresis. This suggests that the relationship between these parameters depends on aronia concentration, the specific stage of addition, and complex matrix interactions. This inconsistency can be attributed to the methodological differences between spontaneous syneresis and centrifugal WHC, as well as the mechanical stress inherent in stirred‐type processing. The breaking of the gel network during stirring, combined with the significant pH drop (3.87) observed in sample K, likely caused excessive contraction of the casein micelles, leading to high spontaneous serum separation (Priyashantha et al. 2025; Yue et al. 2022). However, under the external force of centrifugation, the fiber and pectin content of the aronia puree may have physically trapped the remaining water, which may explain the relatively high WHC value despite the weakened gel structure (Zhang et al. 2024). These findings highlight that physical processing, and the degree of post‐acidification play as critical a role as aronia concentration and probiotic supplementation in modulating yogurt microstructure. Similar to our findings, Du et al. (2023) noted that adding mulberry pulp to yogurt increased WHC. However, some studies reported the opposite outcome: the addition of aronia fiber decreased WHC due to its poor solubility, low water absorption capacity, and tannin‐induced protein precipitation (Szajnar et al. 2021). Moreover, in our study, it was observed that the addition of probiotics increased WHC in yogurts, which may be attributed to the strengthening effect of *L. acidophilus* on the protein network structure (Khalil et al. 2022). This effect is likely linked to the production of exopolysaccharides by the probiotic strain, which interact with milk proteins to reinforce the three‐dimensional matrix and effectively trap water molecules within the gel structure (Korcz and Varga 2021).

Incorporation of aronia puree led to notable alterations in the color parameters of the yogurts (Table 4). As expected, L* values were notably lower in yogurts with aronia compared to the controls, with the greatest decrease observed in samples containing 6% aronia, indicating reduced lightness due to the addition of deeply pigmented phenolic compounds (Adjei et al. 2024; Nguyen and Hwang 2016). While probiotic supplementation had no significant effect on lightness, the overall darkening was attributed to the natural color of aronia. In terms of a* values, the control yogurts exhibited negative values indicating greenish tones, while all aronia supplemented samples exhibited positive a* values reflecting an increase in redness. This enhancement in redness, which almost doubled with increasing aronia concentration, could be attributed to the high anthocyanin and flavonoid content of aronia (Oszmiański and Wojdylo 2005). The addition of aronia puree reduced the b* values in both probiotic and non‐probiotic yogurts, with the decrease in yellowness becoming more pronounced at higher concentrations. This change may be related to the high anthocyanin content of aronia, particularly cyanidin glycosides (e.g., cyanidin‐3‐O‐galactoside), which contribute to the characteristic red‐purple color of the fruit (Kulling and Rawel 2008; Wieloch and Konopacka 2025). Accordingly, these pigments may have contributed to the lower b* values observed in the aronia‐containing yogurts. However, statistically significant increases in b* values were observed in all samples at the end of storage and these findings are consistent with the results reported by Nguyen and Hwang (2016) and Park et al. (2015).

**TABLE 4:** Effects of aronia puree and *L. acidophilus* DSM 20079 addition on L*, a*, and b* color parameters of yogurt samples during storage.

Color parameter	Yogurts^†^	Storage period (day)		
		1	14	28
L*	A	85.14 ± 0.65^B,a^	84.79 ± 0.38^B,a^	85.62 ± 0.76^B,a^
	B	74.55 ± 0.42^D,a^	74.68 ± 0.24^D,a^	75.26 ± 0.76^C,a^
	C	96.84 ± 0.02^A,a^	96.97 ± 0.09^A,a^	96.77 ± 0.27^A,a^
	D	96.03 ± 0.85^A,a^	97.05 ± 0.04^A,a^	97.23 ± 0.13^A,a^
	E	85.12 ± 0.09^B,ab^	84.80 ± 0.15^B,b^	85.55 ± 0.14^B,a^
	F	75.10 ± 0.07^D,a^	74.76 ± 0.68^D,a^	74.50 ± 0.29^C,a^
	K	82.97 ± 0.84^C,a^	83.40 ± 0.23^C,a^	84.33 ± 0.12^B,a^
	L	71.86 ± 0.16^E,a^	71.70 ± 0.12^E,a^	72.20 ± 0.07^D,a^
a*	A	8.32 ± 0.14^D,a^	8.13 ± 0.04^D,a^	6.94 ± 0.21^D,b^
	B	16.40 ± 0.24^B,a^	16.33 ± 0.14^B,ab^	15.57 ± 0.17^B,b^
	C	−5.14 ± 0.00^E,a^	−5.10 ± 0.01^E,a^	−5.09 ± 0.02^E,a^
	D	−4.91 ± 0.23^E,a^	−5.14 ± 0.02^E,a^	−5.11 ± 0.05^E,a^
	E	7.95 ± 0.17^D,a^	7.49 ± 0.10^D,a^	6.68 ± 0.06^D,b^
	F	16.42 ± 0.34^B,a^	16.10 ± 0.11^B,a^	15.82 ± 0.07^B,a^
	K	9.90 ± 0.36^C,a^	9.26 ± 0.32^C,ab^	8.29 ± 0.00^C,b^
	L	19.11 ± 0.03^A,a^	19.63 ± 0.27^A,a^	18.78 ± 0.18^A,a^
b*	A	9.50 ± 0.03^C,c^	10.20 ± 0.04^C,b^	10.53 ± 0.07^B,a^
	B	7.32 ± 0.07^DE,b^	8.50 ± 0.18^E,a^	8.66 ± 0.07^C,a^
	C	15.64 ± 0.07^A,b^	16.04 ± 0.01^A,ab^	16.45 ± 0.29^A,a^
	D	13.89 ± 1.13^B,a^	15.26 ± 0.07^B,a^	16.06 ± 0.19^A,a^
	E	9.20 ± 0.02^C,c^	10.04 ± 0.04^C,b^	10.41 ± 0.12^B,a^
	F	7.23 ± 0.05^DE,c^	7.92 ± 0.03^F,b^	8.15 ± 0.06^C,a^
	K	8.44 ± 0.17^CD,c^	9.26 ± 0.07^D,b^	10.09 ± 0.07^B,a^
	L	6.51 ± 0.27^E,b^	7.85 ± 0.24^F,a^	7.96 ± 0.32^C,a^

*Note:* Different uppercase letters within the same column indicate significant differences among samples, while different lowercase letters within the same row indicate significant differences during storage (*p* < 0.05).

^†^ Yogurt A, set‐type yogurt formulated with 2% aronia puree and *L. acidophilus* DSM 20079; Yogurt B, set‐type yogurt formulated with 6% aronia puree and *L. acidophilus* DSM 20079; Yogurt C, set‐type control yogurt formulated with *L. acidophilus* DSM 20079 and without aronia puree; Yogurt D, set‐type control yogurt formulated without aronia puree and *L. acidophilus* DSM 20079; Yogurt E, set‐type yogurt formulated with 2% aronia puree and without *L. acidophilus* DSM 20079; Yogurt F, set‐type yogurt formulated with 6% aronia puree and without *L. acidophilus* DSM 20079; Yogurt K, stirred‐type yogurt formulated with 2% aronia puree; Yogurt L, stirred‐type yogurt formulated with 6% aronia puree.

### 3.2 Hardness of Yogurts

**FIGURE 4:**
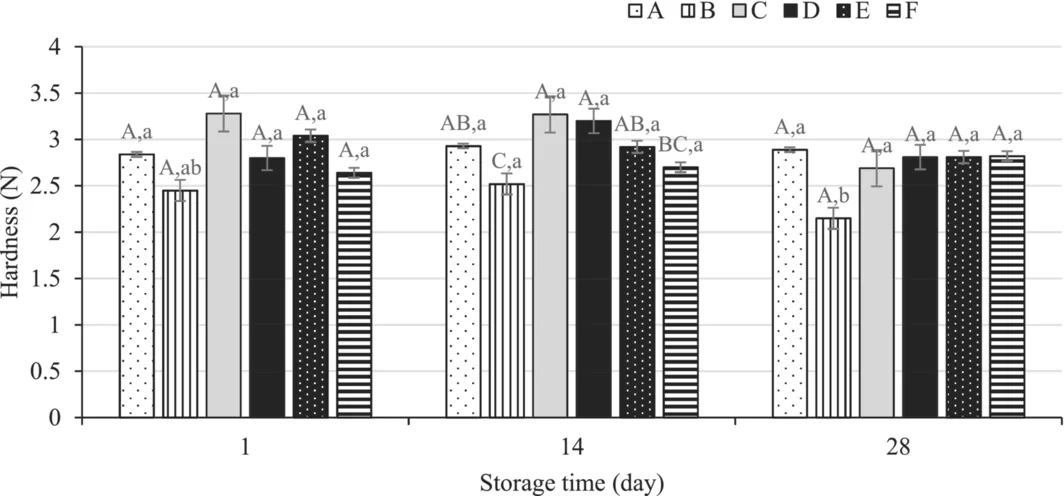
Changes in yogurt hardness during storage. Different uppercase letters above the bars indicate significant differences among yogurt samples, whereas different lowercase letters indicate significant differences during storage (*p* < 0.05). Yogurt A, set‐type yogurt formulated with 2% aronia puree and *L. acidophilus* DSM 20079; Yogurt B, set‐type yogurt formulated with 6% aronia puree and *L. acidophilus* DSM 20079; Yogurt C, set‐type control yogurt formulated with *L. acidophilus* DSM 20079 and without aronia puree; Yogurt D, set‐type control yogurt formulated without aronia puree and *L. acidophilus* DSM 20079; Yogurt E, set‐type yogurt formulated with 2% aronia puree and without *L. acidophilus* DSM 20079; Yogurt F, set‐type yogurt formulated with 6% aronia puree and without *L. acidophilus* DSM 20079.

The effects of aronia puree and probiotic culture incorporation on the hardness of set‐type yogurts are presented in Figure 4. No statistically significant differences in hardness values were observed upon aronia and probiotic addition at the beginning of storage. On Day 14 of storage, the hardness of both control yogurts, with and without probiotics, exhibited the highest values. Moreover, the addition of aronia significantly reduced the hardness values (*p* < 0.05), with a further decline observed as the concentration of the aronia increased, indicating a weakening of the gel structure. Yogurt hardness reflects the strength of the gel network, with higher hardness values indicating a firmer and more compact gel structure (Feng et al. 2019). Similarly, comparable trends have been reported in set‐type yogurts with fruit; for instance, Feng et al. (2019) demonstrated that the firmness of yogurt decreased significantly with the increasing addition of jujube puree. Collectively, these findings indicate that aronia puree reduced yogurt hardness in a concentration‐dependent manner, whereas probiotic culture did not significantly affect hardness during storage.

### 3.3 Microbiological Evaluation

**FIGURE 5:**
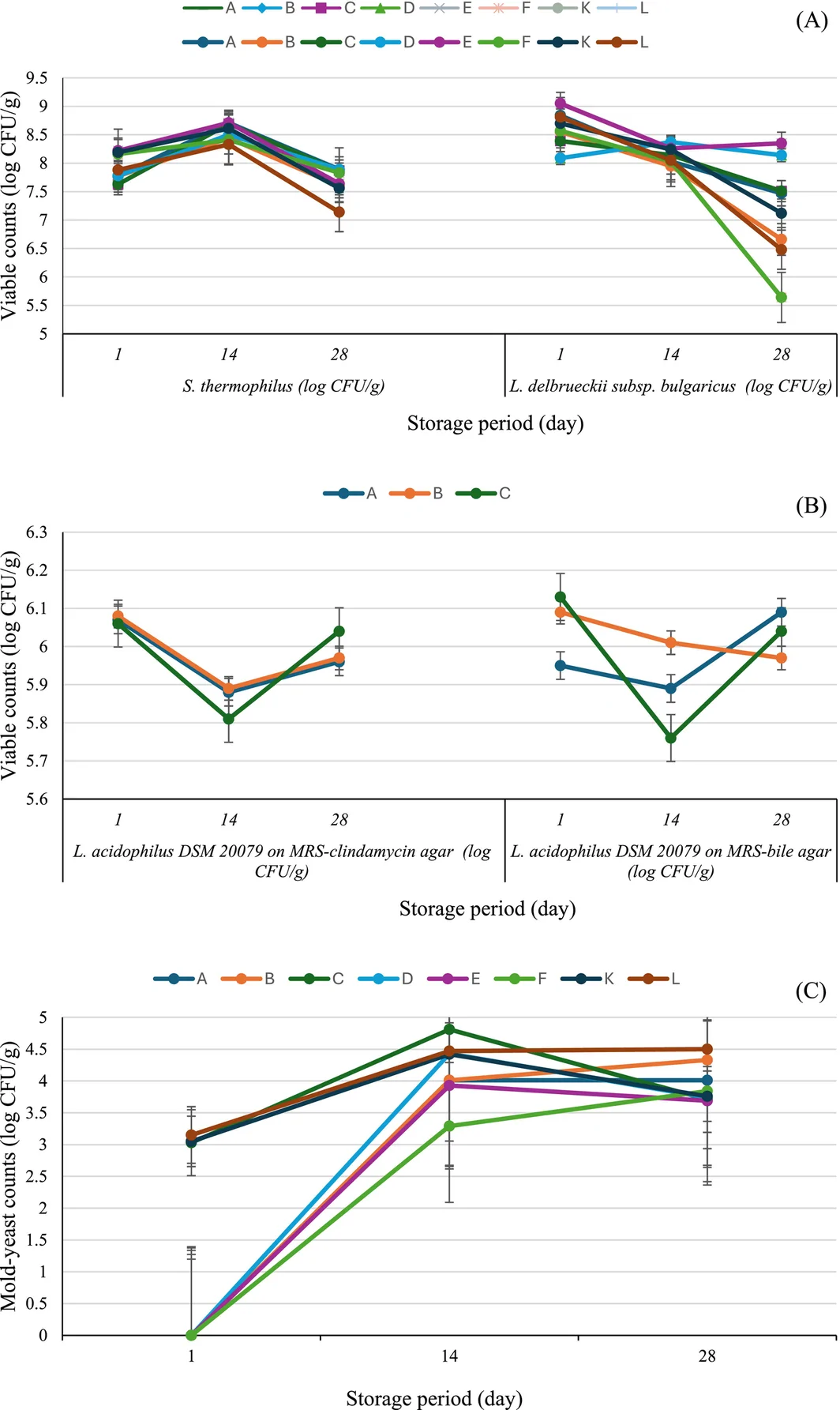
Viability of *S. thermophilus* , *L. delbrueckii* subsp. *bulgaricus* (A), *L. acidophilus* DSM 20079 (B), and yeasts and molds (C) counts (log CFU/g) of yogurt samples during storage. Yogurt A, set‐type yogurt formulated with 2% aronia puree and *L. acidophilus* DSM 20079; Yogurt B, set‐type yogurt formulated with 6% aronia puree and *L. acidophilus* DSM 20079; Yogurt C, set‐type control yogurt formulated with *L. acidophilus* DSM 20079 and without aronia puree; Yogurt D, set‐type control yogurt formulated without aronia puree and *L. acidophilus* DSM 20079; Yogurt E, set‐type yogurt formulated with 2% aronia puree and without *L. acidophilus* DSM 20079; Yogurt F, set‐type yogurt formulated with 6% aronia puree and without *L. acidophilus* DSM 20079; Yogurt K, stirred‐type yogurt formulated with 2% aronia puree; Yogurt L, stirred‐type yogurt formulated with 6% aronia puree.

The levels of *S. thermophilus* , *L. delbrueckii* subsp. *bulgaricus*, *L. acidophilus* DSM 20079, as well as yeasts and molds, were monitored in yogurts over a 28‐day storage period (Figure 5). On the first day of storage, *S. thermophilus* counts ranged from 7.63 to 8.22 log CFU/g, with no statistically significant differences observed between samples with or without aronia and probiotic supplementation. By Day 14, a significant increase in viable counts of *S. thermophilus* was detected in all samples (*p* < 0.05), with the highest increase (1.06 log CFU/g) observed in yogurt C, while the lowest increases were recorded in yogurt K (0.42 log CFU/g) and yogurt L (0.45 log CFU/g). Although counts declined significantly by Day 28, they remained comparable to those on Day 1, consistent with previous reports describing an initial increase followed by a gradual decrease during storage (Dimitrellou et al. 2020; Shori et al. 2022). Maintaining the viability of *S. thermophilus* in yogurts during storage is therefore essential not only for ensuring product quality but also for supporting its suggested probiotic functionality (Tarrah et al. 2018; Uriot et al. 2017).

The highest initial count of *L. delbrueckii* subsp. *bulgaricus* was recorded in sample E (9.05 log CFU/g). Although viable counts declined during storage, sample E retained the highest population on Day 28 (8.35 log CFU/g), whereas sample F showed the greatest reduction (5.64 log CFU/g). Similar decreases were also observed in stirred‐type yogurts K and L. This decline may be attributed to microbial stress induced by the late exposure to phenolic compounds (Rogalska et al. 2024). These results suggest that various factors, such as antioxidant activity or other antimicrobial agents in aronia, may exert a concentration‐dependent effect on microbial viability, with lower concentrations promoting microbial growth and higher concentrations exhibiting inhibitory activity (Bontsidis et al. 2021). Moreover, the addition of probiotic cultures did not exhibit a direct, statistically significant influence on *L. bulgaricus* viability. Overall, the outcomes indicate that prior to fermentation, supplementation with aronia at lower concentrations is more effective in preserving *L. bulgaricus* viability and offers a more favorable condition for microbial stability.

In probiotic yogurts, viable counts of *L. acidophilus* DSM 20079, enumerated using two distinct selective media—MRS agar supplemented with clindamycin and MRS agar supplemented with bile salts—remained above the threshold level of 6 log CFU/g, which is considered sufficient for probiotic functionality (Champagne et al. 2011). No significant differences were observed between the two media, confirming that both were equally effective for viable cell enumeration (*p* > 0.05). Likewise, aronia supplementation had no statistically significant effect on the viability of *L. acidophilus* DSM 20079. This finding aligned with previous reports indicating that phenol‐rich plant extracts, such as grape pomace and apple peel extracts, did not adversely influence probiotic survival in fermented dairy products (Ahmad et al. 2020; Dos Santos et al. 2017). The lack of significant differences between yogurts containing 2% and 6% aronia puree further suggests that the phenolic compounds naturally present in aronia did not adversely affect the viability of *L. acidophilus* DSM 20079 under the conditions of this study. This observation is also consistent with studies showing that certain probiotic strains can maintain their viability in dairy matrices enriched with polyphenol‐rich plant ingredients (Ahmad et al. 2020). In addition, the yogurt matrix may have provided a protective environment that supported probiotic survival during refrigerated storage, despite the presence of phenolic compounds (Plessas et al. 2023). Considering the high phenolic content of aronia, these findings suggest that aronia puree can be incorporated into probiotic yogurt without compromising the viability of *L. acidophilus* DSM 20079. Yeast and mold counts remained below the limit stipulated by the Turkish Food Codex in some samples on the first day of storage but increased significantly in all samples in the following days (Turkish Food Codex 2022). However, no statistically significant differences were observed between samples on Days 14 and 28. These findings suggest that the addition of aronia puree and probiotic cultures had a limited effect on yogurt microbiota while changes in yeast and mold counts were primarily associated with storage time.

### 3.4 α‐Glucosidase and ACE Inhibitory Activity of Yogurt Samples

**FIGURE 6:**
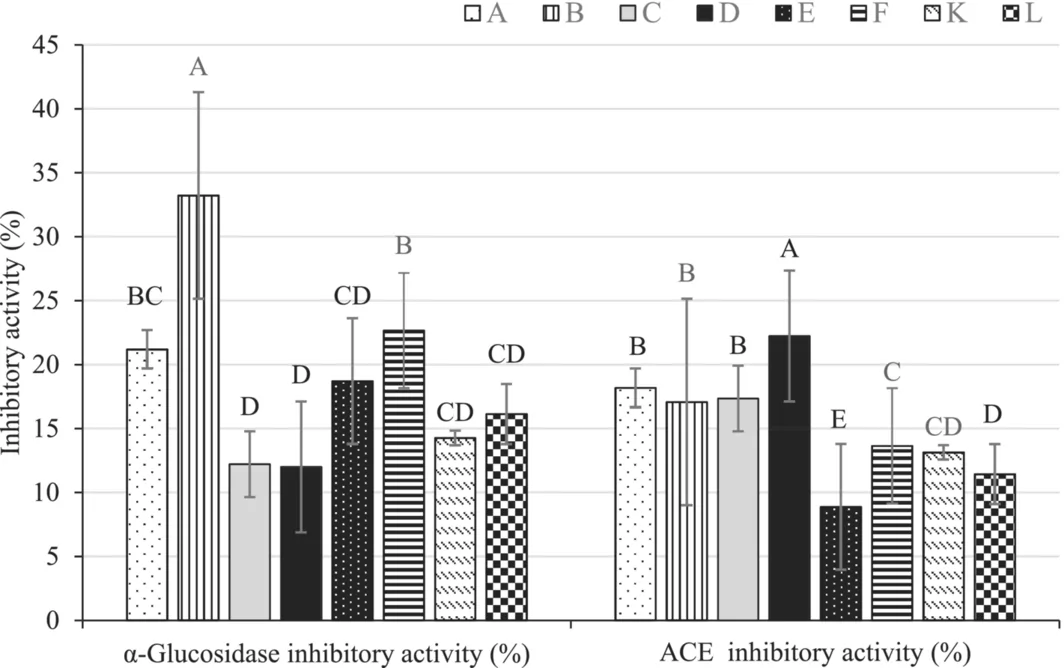
α‐Glucosidase and angiotensin‐converting enzyme (ACE) inhibitory activities of yogurt samples on the 28th day of refrigerated storage. Different letters above the bars indicate significant differences among samples (*p* < 0.05). (A) Set‐type yogurt formulated with 2% aronia puree and *L. acidophilus* DSM 20079; (B) Set‐type yogurt formulated with 6% aronia puree and *L. acidophilus* DSM 20079; (C) Set‐type control yogurt formulated with *L. acidophilus* DSM 20079 and without aronia puree; (D) Set‐type control yogurt formulated without aronia puree and *L. acidophilus* DSM 20079; (E) Set‐type yogurt formulated with 2% aronia puree and without *L. acidophilus* DSM 20079; (F) Set‐type yogurt formulated with 6% aronia puree and without *L. acidophilus* DSM 20079; (K) Stirred‐type yogurt formulated with 2% aronia puree; (L) Stirred‐type yogurt formulated with 6% aronia puree.

α‐Glucosidase inhibitors, particularly fruit‐derived polyphenols, can help reduce postprandial glucose levels and may offer natural strategies for Type 2 diabetes management (Pyner et al. 2017; Zahid et al. 2022). The α‐glucosidase inhibition activity of yogurts measured on Day 28 of storage is demonstrated in Figure 6. The findings of this study revealed that adding aronia significantly increased the α‐glucosidase inhibitory activity of yogurt. This effect was further enhanced by the addition of the probiotic *L. acidophilus* DSM 20079. Yogurt B (6% aronia+probiotics) exhibited the highest α‐glucosidase inhibition activity (33.23%), whereas the control yogurt C showed the lowest level (12.21%) (*p* < 0.05). Regardless of the probiotic content, all aronia‐supplemented samples showed significantly higher α‐glucosidase inhibitory activity compared to control groups, indicating that aronia‐derived phenolic compounds have a strong inhibitory potential against the enzyme (Imai et al. 2020). Increasing the aronia concentration from 2% to 6% resulted in a 56.74% increase in α‐glucosidase inhibition in yogurts containing probiotics; however, this increase remained limited to 21.11% in non‐probiotic samples. These results suggest a synergistic interaction between aronia polyphenols and probiotic cultures in enhancing α‐glucosidase inhibition. This synergy is likely associated with the ability of probiotics to produce bioactive compounds such as exopolysaccharides and bioactive peptides exhibiting α‐glucosidase inhibitory properties (Muganga et al. 2015; Zeng et al. 2016). Indeed, previous studies reported that yogurts enriched with polysaccharides exhibited higher inhibition capacity compared to polysaccharide‐free formulations, and this effect was attributed to exopolysaccharides synthesized by lactobacilli (Ramchandran and Shah 2009). Moreover, significantly lower inhibition rates were observed in yogurts to which aronia was added after fermentation (*p* < 0.05). This suggests that the higher inhibitory activity in set‐type yogurts may be linked to the presence of aronia during fermentation, which could promote the formation of metabolites, such as exopolysaccharides. In line with these findings, Zahid et al. (2022) also reported that yogurts containing both mango peel powder and probiotics exhibited greater α‐glucosidase inhibition than those containing only fruit powder. These results collectively highlight the potential of combining aronia and probiotics in the development of functional dairy products aimed at supporting the dietary management of metabolic disorders such as Type 2 diabetes.

ACE inhibitors prevent vasoconstriction and lower blood pressure by blocking the production of angiotensin II (Stuknytė et al. 2015). Therefore, the use of ACE inhibitors is recognized as an effective treatment for hypertension (Shu et al. 2015). Fermented milk represents a rich matrix for the production of bioactive peptides, especially ACE inhibitory peptides, whose generation depends on variables such as starter culture, substrate composition, and processing conditions (Du et al. 2023). As shown in Figure 6, the control yogurt D (without probiotic) exhibited the highest ACE inhibitory activity at 22.23%. On the other hand, the ACE inhibitory activities of aronia‐enriched yogurts without probiotics (samples E and F) were significantly lower than the control (*p* < 0.05). This finding may be associated with reduced formation of bioactive peptides resulting from interactions between milk proteins and aronia polyphenols. (Kim, Lee, et al. 2021). Furthermore, excess phenolic compounds may have inhibited growth of lactic acid bacteria and proteolytic systems, leading to a decrease in peptide production and hence reduced ACE‐I activity of yogurt (Du et al. 2023). In agreement with the present study, Du et al. (2023) demonstrated a concentration‐dependent decrease in ACE inhibitory activity in set‐type yogurts fortified with mulberry pomace, while Terzioğlu et al. (2023) reported a similar inhibitory effect in buffalo milk yogurts supplemented with strawberry puree.

When probiotic‐free yogurt samples with the same aronia concentrations were compared, no consistent trend of increase or decrease in ACE inhibitory activity was observed between set‐ (E and F) and stirred‐type (K and L) yogurts. This indicated that the relationship between yogurt type and aronia concentration does not result in a linear or predictable effect on ACE inhibitory activity. Therefore, based on the available data, it is not possible to establish a systematic effect model regarding aronia concentration and the addition stage for both yogurt types. Probiotic yogurts containing aronia (A and B) exhibited higher ACE inhibitory activity compared to non‐probiotic set‐type yogurts (E and F), suggesting a synergistic effect between the probiotic strain *L. acidophilus* DSM 20079 and aronia constituents on bioactive peptide formation. These results agreed with previous findings suggesting that probiotics enhance the generation of antihypertensive peptides through their proteolytic activities (Kim, Park, and Kim 2021). Cell wall‐bound proteinases of microorganisms used in the fermentation process show broad substrate specificity, which explains the potential contribution of probiotics in ACE inhibitory peptide production (Sebastián‐Nicolas et al. 2021). Supporting this, Shakerian et al. (2015) reported that probiotic yogurts with inulin demonstrated significantly higher ACE inhibitory activity compared to non‐probiotic yogurts with inulin. These findings suggest that the combined presence of probiotics and aronia in yogurt may enhance its antihypertensive potential, likely through increased release of bioactive peptides during fermentation (Kayacan Çakmakoğlu et al. 2024).

### 3.5 Effects of In Vitro Gastrointestinal Digestion on TPC and Antioxidant Capacity of Yogurt Samples

Table 5 summarizes the TPC and antioxidant activity (measured by DPPH and FRAP methods) of yogurt samples on day 28 of storage, as well as their changes simulated in vitro digestion through the gastric and intestinal phases. The TPC of the undigested probiotic control sample was slightly decreased by the addition of 2% aronia (sample A), although this reduction was not statistically significant (*p* > 0.05). Comparable decreases in TPC have been reported in yogurts supplemented with pomegranate juice powder (Pan et al. 2019) and grape‐callus extracts (Karaaslan et al. 2011). This effect may be attributed to protein–polyphenol complex formation within the yogurt matrix (Ozdal et al. 2013; Vital et al. 2015). Conversely, yogurt B (6% aronia + probiotic) exhibited a significant increase in TPC, representing a 71.04% enhancement compared to the control (*p* < 0.05). This finding was consistent with previous reports demonstrating the positive effect of higher aronia concentrations on yogurt phenolic content (Dimitrellou et al. 2020; Plessas et al. 2023). The observed enhancement is consistent with previous reports describing *A. melanocarpa* as a rich source of phenolic acids, flavonols, anthocyanins, and proanthocyanidins, compounds that have been associated with its high phenolic content and antioxidant potential (Gao et al. 2023). Although the total phenolic content of the aronia puree was not directly determined in this study, *A. melanocarpa* is widely reported as a rich source of phenolic compounds, with levels typically ranging from 9.20 mg GAE/g to 25.6 mg GAE/g fresh weight depending on the cultivar and maturity stage (Jurikova et al. 2017; Yuan et al. 2018). While thermal treatment (80°C for 10 min) initially causes some degradation of heat‐labile phenolics (Repajić et al. 2025), the significant 71.04% increase in TPC observed in yogurt B compared to the control (Table 5) confirms that a substantial concentration of aronia phenolics was successfully retained. This high retention is likely attributed to the protective effect of milk proteins, which form complexes with polyphenols, thereby providing matrix protection against thermal and environmental stressors during processing and storage (Lamothe et al. 2014; Plessas et al. 2023). Moreover, neither the addition stage (pre‐ or post‐fermentation) of aronia puree nor probiotic supplementation significantly influenced TPC levels (*p* > 0.05).

**TABLE 5:** Total phenolic content, antioxidant activity, and their changes during simulated digestion in yogurt samples.

	Yogurts^†^	Undigested	In vitro post digestion	
			Gastric phase	Intestinal phase
Total phenolic content (mg GAE/100 g)	A	105.50 ± 17.70^C,c^	314.00 ± 19.80^AB,b^	528.80 ± 12.45^A,a^
	B	209.70 ± 1.56^A,b^	376.00 ± 6.79^A,b^	702.40 ± 81.50^A,a^
	C	122.60 ± 0.56^bc,b^	196.80 ± 20.40^C,b^	648.00 ± 32.80^A,a^
	D	113.80 ± 0.56^C,b^	175.60 ± 11.88^C,b^	614.80 ± 25.50^A,a^
	E	153.70 ± 4.67^B,b^	197.60 ± 28.30^C,b^	684.00 ± 66.80^A,a^
	F	210.30 ± 15.40^A,b^	253.00 ± 16.10^bc,b^	692.00 ± 27.20^A,a^
	K	134.80 ± 0.28^bc,b^	222.80 ± 37.90^bc,b^	676.00 ± 65.60^A,a^
	L	209.90 ± 3.25^A,b^	242.00 ± 45.80^B,b^	709.60 ± 63.40^A,a^
DPPH (μM TEAC/mg)	A	24.06 ± 3.56^B,a^	4.60 ± 0.46^DE,b^	5.58 ± 1.75^C,b^
	B	53.79 ± 4.18^A,a^	8.23 ± 0.20^CD,b^	17.32 ± 1.31^A,b^
	C	10.45 ± 0.62^B,a^	3.25 ± 0.00^E,b^	5.73 ± 1.31^C,b^
	D	21.91 ± 1.99^B,a^	8.75 ± 1.46^CD,b^	8.13 ± 2.08^C,b^
	E	22.73 ± 1.67^B,a^	13.63 ± 0.73^B,b^	8.12 ± 0.32^C,c^
	F	55.12 ± 5.65^A,a^	24.06 ± 2.34^A,b^	13.61 ± 1.31^AB,b^
	K	22.03 ± 1.52^B,a^	12.00 ± 1.78^bc,b^	9.05 ± 0.76^bc,b^
	L	44.33 ± 5.02^A,a^	20.06 ± 0.20^A,b^	16.31 ± 0.54^A,b^
FRAP (mg Trolox/100 g)	A	9.47 ± 1.51^A,b^	15.60 ± 1.21^bc,b^	47.45 ± 2.62^E,a^
	B	10.11 ± 1.21^A,b^	17.31 ± 3.64^bc,b^	54.17 ± 2.43^DE,a^
	C	9.04 ± 0.30^A,c^	17.52 ± 1.11^bc,b^	58.23 ± 1.53^D,a^
	D	8.61 ± 0.30^A,c^	19.88 ± 0.80^bc,b^	73.88 ± 1.21^AB,a^
	E	7.97 ± 0.00^A,c^	22.45 ± 0.80^B,b^	68.74 ± 3.64^bc,a^
	F	10.33 ± 0.30^A,c^	34.46 ± 3.64^A,b^	61.03 ± 2.42^CD,a^
	K	7.54 ± 0.60^A,c^	13.88 ± 1.21^C,b^	71.31 ± 0.00^B,a^
	L	9.25 ± 0.60^A,b^	15.60 ± 1.21^bc,b^	81.60 ± 2.42^A,a^

*Note:* Different uppercase letters within the same column indicate significant differences among samples, while different lowercase letters within the same row indicate significant differences during storage (*p* < 0.05).

^†^ Yogurt A, set‐type yogurt formulated with 2% aronia puree and *L. acidophilus* DSM 20079; Yogurt B, set‐type yogurt formulated with 6% aronia puree and *L. acidophilus* DSM 20079; Yogurt C, set‐type control yogurt formulated with *L. acidophilus* DSM 20079 and without aronia puree; Yogurt D, set‐type control yogurt formulated without aronia puree and *L. acidophilus* DSM 20079; Yogurt E, set‐type yogurt formulated with 2% aronia puree and without *L. acidophilus* DSM 20079; Yogurt F, set‐type yogurt formulated with 6% aronia puree and without *L. acidophilus* DSM 20079; Yogurt K, stirred‐type yogurt formulated with 2% aronia puree; Yogurt L, stirred‐type yogurt formulated with 6% aronia puree.

In vitro analyses conducted on the 28th day of storage showed that the free radical scavenging activity evaluated by the DPPH method increased by 130.15% and 414.93% in yogurt A and yogurt B, respectively, compared to the yogurt C (Table 5). This may be attributed to the high levels of biologically active polyphenols in aronia, such as anthocyanins (e.g., cyanidin‐3‐galactoside), proanthocyanidins, and flavonols, which are associated with free radical scavenging capacity (Dobros et al. 2024). Neither the aronia addition stage nor the use of probiotics significantly affected the DPPH radical scavenging activity of the yogurts. In contrast, the reduction capacity determined by the FRAP method showed a limited increase in all yogurts with aronia compared to the control groups, but these differences were not statistically significant (*p* > 0.05). The different responses of the DPPH and FRAP assays may be attributable to their distinct reaction mechanisms (Yilmaz‐Ersan et al. 2024). While the DPPH assay primarily evaluates free radical scavenging activity, the FRAP assay measures ferric ion reducing capacity (Rogalska et al. 2024). In addition, interactions between aronia polyphenols and milk proteins may limit the iron‐reducing response, resulting in a smaller increase in FRAP values (Feng et al. 2023; Yu et al. 2023). This difference is consistent with findings by Nguyen and Hwang (2016) in aronia‐fortified yogurts, who found that DPPH activity increased with aronia concentration, whereas no significant changes were observed in FRAP values.

The simulated gastrointestinal digestion exhibited significant effects on the TPC and antioxidant capacity of the yogurts (Table 5). During the gastric phase, TPC levels increased significantly, with a 91.05% rise observed in the probiotic yogurt enriched with 6% aronia (*p* < 0.05). Meanwhile, yogurt F (6% aronia without probiotic) showed a 44.07% increase compared to the corresponding control in the gastric phase. This suggests that the enhanced TPC may be attributed not only to the addition of aronia but also to the probiotic cultures, which are known to improve the bioaccessibility of phenolic compounds in various food matrices (Hole et al. 2012; Valero‐Cases et al. 2017). In the intestinal phase, all samples exhibited higher TPC values compared to both the initial and gastric phases. This enhancement may be attributed to the release of phenolic compounds from protein–phenolic complexes during digestion (Helal and Tagliazucchi 2018). In contrast, DPPH values decreased throughout digestion, with significant losses detected particularly in the gastric phase (*p* < 0.05), likely due to the degradation of polyphenols under the acidic conditions of the stomach (Lorrain et al. 2012). Contrary to DPPH findings, FRAP values increased post‐gastric digestion and peaked in the intestinal phase across all yogurt formulations. For instance, in the intestinal phase, the FRAP value of yogurt containing 6% aronia increased by 435.80% compared to the undigested state, suggesting enhanced polyphenol bioaccessibility due to matrix protection during digestion (Lamothe et al. 2014). These contrasting trends likely reflect the different analytical characteristics of the DPPH and FRAP assays. DPPH appears to be more susceptible to pH‐induced degradation of antioxidant compounds, whereas FRAP more effectively reflects the increased electron‐donating capacity observed after gastrointestinal digestion (Lang et al. 2024). Overall, aronia and probiotic supplementation enhanced the TPC and antioxidant capacity of yogurts before digestion; these effects became more pronounced during the simulated intestinal phase, indicating increased bioaccessibility and functional potential.

### 3.6 Evaluation of Cytotoxic Effects of Yogurts

The cytotoxic effects of probiotic supplementation and aronia enrichment in set‐ and stirred‐type yogurt samples on the human colon cancer cell line (Caco‐2) are presented in Table 6. Yogurt samples were prepared at 3% and 5% (v/v) concentrations and incubated with Caco‐2 cells for 24 and 48 h. Following 24 h of incubation, at 3% concentration, the lowest cell viability was observed in yogurt sample K, while the highest was in sample L. Although no statistically significant difference was found among the groups, yogurt K exhibited the strongest anticancer potential by reducing cell viability by 20.40%. At 5% concentration, all samples showed comparable cell viability with no significant differences (*p* > 0.05). Viability increased significantly with higher concentrations, indicating no cytotoxic effect on Caco‐2 cells after 24 h incubation. After 48 h of incubation, no statistically significant differences in cell viability were found at either 3% or 5% concentrations (Table 6). However, at 3%, an increase in cell viability was observed in all samples except A and L, with the highest increase detected in yogurt F (32.21%). Conversely, extending the incubation period to 48 h at 5% concentration led to a decrease in cell viability in all samples, with the greatest reduction in sample F (37.90%) and the lowest in sample L (24.79%). These findings suggest that the cytotoxic activity of yogurt samples may depend on both concentration and exposure time. Previous studies have demonstrated the cytotoxic effects of yogurt and its derivatives on various cancer cell lines, highlighting the role of yogurt composition in modulating these effects (Ciric et al. 2023; de Moreno and Perdigon 2004; Parvarei and Mortazavian 2022). Although the cytotoxic potential of aronia against cancer cells has been well documented (Cvetanović et al. 2018; Gürer and Altıntaş 2024; Kim, Park, and Kim 2021), the present study found that this activity decreased when aronia was incorporated into a yogurt matrix. This reduction may be attributed to the low pH and specific enzymatic composition of yogurt, particularly the proteases and peptidases produced by the starter and probiotic cultures during fermentation (Kim, Lee, et al. 2021). Furthermore, aronia fruit provides usable sugars, including glucose, fructose, and sorbitol (Kulling and Rawel 2008), which support the metabolic activity of these microorganisms (Nguyen and Hwang 2016). The resulting microbial activity and enzyme production can alter the structural integrity and function of aronia‐derived phenolic compounds (Du and Myracle 2018b; Efenberger‐Szmechtyk et al. 2020). Moreover, interactions between yogurt proteins and aronia polyphenols could reduce the bioavailability and biological activity of these compounds (Bokov et al. 2022; Efenberger‐Szmechtyk et al. 2020). These results suggest that the incorporation of functional compounds into food matrices may significantly influence their biological efficacy. Further research may contribute to a better understanding of these mechanisms.

**TABLE 6:** Cytotoxic effects of yogurt samples on human colorectal cancer cell line (Caco‐2).

Yogurts^†^	Cell viability (%) after a 24 h incubation period		Cell viability (%) after a 48 h incubation period	
	3% concentration	5% concentration	3% concentration	5% concentration
A	102.38 ± 3.15^AB,b^	173.00 ± 10.96^A,a^	96.42 ± 11.85^A,a^	112.40 ± 3.99^A,a^
B	103.57 ± 13.24^AB,b^	169.72 ± 4.98^A,a^	109.05 ± 0.63^A,a^	113.08 ± 1.75^A,a^
C	96.58 ± 5.88^AB,b^	140.38 ± 2.66^A,a^	109.27 ± 1.58^A,a^	104.74 ± 5.90^A,a^
D	99.85 ± 1.26^AB,b^	166.43 ± 2.32^A,a^	107.48 ± 0.31^A,b^	125.02 ± 1.11^A,a^
E	97.92 ± 3.99^AB,b^	168.54 ± 7.97^A,a^	122.90 ± 0.31^A,a^	123.45 ± 4.94^A,a^
F	88.56 ± 1.68^AB,b^	188.26 ± 13.28^A,a^	117.09 ± 2.84^A,a^	116.90 ± 14.50^A,a^
K	79.60 ± 15.10^B,a^	147.90 ± 21.20^A,a^	97.00 ± 17.10^A,a^	116.01 ± 3.35^A,a^
L	114.41 ± 10.51^A,a^	165.50 ± 20.30^A,a^	110.50 ± 8.37^A,a^	124.46 ± 12.12^A,a^

*Note:* Different uppercase letters within the same column indicate significant differences among samples, while different lowercase letters within the same row indicate significant differences during storage (*p* < 0.05).

^†^ Yogurt A, set‐type yogurt formulated with 2% aronia puree and *L. acidophilus* DSM 20079; Yogurt B, set‐type yogurt formulated with 6% aronia puree and *L. acidophilus* DSM 20079; Yogurt C, set‐type control yogurt formulated with *L. acidophilus* DSM 20079 and without aronia puree; Yogurt D, set‐type control yogurt formulated without aronia puree and *L. acidophilus* DSM 20079; Yogurt E, set‐type yogurt formulated with 2% aronia puree and without *L. acidophilus* DSM 20079; Yogurt F, set‐type yogurt formulated with 6% aronia puree and without *L. acidophilus* DSM 20079; Yogurt K, stirred‐type yogurt formulated with 2% aronia puree; Yogurt L, stirred‐type yogurt formulated with 6% aronia puree.

### 3.7 Sensory Evaluation

**FIGURE 7:**
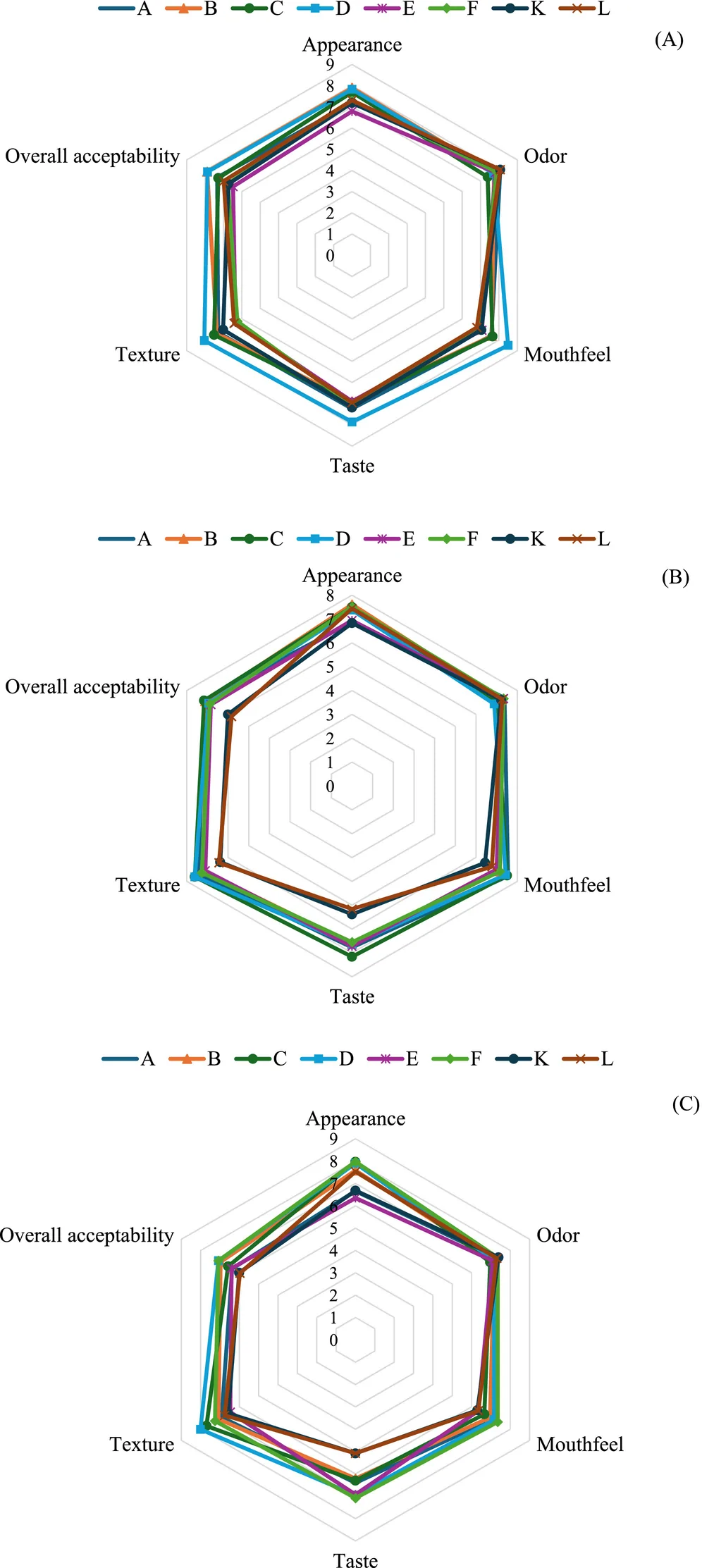
Sensory profiles of yogurts during 1st (A), 14th (B) and 28th (C) day of storage. Yogurt A, set‐type yogurt formulated with 2% aronia puree and *L. acidophilus* DSM 20079; Yogurt B, set‐type yogurt formulated with 6% aronia puree and *L. acidophilus* DSM 20079; Yogurt C, set‐type control yogurt formulated with *L. acidophilus* DSM 20079 and without aronia puree; Yogurt D, set‐type control yogurt formulated without aronia puree and *L. acidophilus* DSM 20079; Yogurt E, set‐type yogurt formulated with 2% aronia puree and without *L. acidophilus* DSM 20079; Yogurt F, set‐type yogurt formulated with 6% aronia puree and without *L. acidophilus* DSM 20079; Yogurt K, stirred‐type yogurt formulated with 2% aronia puree; Yogurt L, stirred‐type yogurt formulated with 6% aronia puree.

Sensory scores of set‐ and stirred‐type yogurts with aronia and *L. acidophilus* DSM 20079 during 28‐day storage are shown in Figure 7. For appearance, no significant differences were observed among the samples on Day 1 (*p* > 0.05); however, by Day 14, set‐type yogurt B (6% aronia+probiotic) received the highest score (7.61), indicating enhanced visual appeal. Aronia is rich in anthocyanins, which impart a purple hue and enhance yogurt color (Nour 2022). By Day 14, increased titratable acidity may have stabilized anthocyanins, contributing to the intensified color and highest appearance score observed in sample B (Chen et al. 2023). At the end of storage, a noticeable decrease in appearance scores was observed in yogurts with 2% aronia (A, E, and K), suggesting that lower concentrations may result in reduced color stability over time (Ścibisz et al. 2019). In terms of odor, all samples initially exhibited high scores (7.39–8.07), but a significant reduction was observed in certain formulations during storage, particularly in plain yogurts D (without probiotic) and C (with probiotic) on Day 14 (D: 6.88, C: 7.00). Regarding mouthfeel, sample D consistently received the highest scores throughout storage, while stirred‐type yogurts K and L recorded the lowest scores by Day 28. These findings suggest that the stage of aronia addition significantly impacts textural perception in the mouth, with post‐fermentation enrichment potentially compromising mechanical consistency. The addition of aronia or probiotics in set‐ and stirred‐type yogurts did not cause significant differences in taste among the samples on the first day of storage (*p* > 0.05). For taste, samples K and L again showed significantly lower acceptability on Days 14 and 28 (both 5.08 on Day 28), revealing that post‐fermentation aronia supplementation may negatively affect flavor. In the texture evaluation, control yogurt D achieved the highest texture scores throughout storage, suggesting that the absence of additives preserved its structural quality. In contrast, stirred‐type yogurts K and L showed reduced texture scores. This situation can be explained by the fact that breaking up the curd after the formation of the gel and adding aronia purée disrupts the casein network, thereby weakening its structural integrity (Priyashantha et al. 2025). In terms of overall acceptability, a similar trend was observed, with K and L yogurts receiving the lowest scores on Day 28, while sample B retained relatively high panelist preference. Also, yogurts without probiotics and with added aronia puree (E and F) received lower scores than those containing probiotics. Aronia fruit is generally characterized by a bitter or astringent taste (Duffy et al. 2016). However, as demonstrated by our findings, the addition of aronia puree did not adversely affect the taste, mouthfeel, or odor of the yogurt samples. Similarly, Nguyen and Hwang (2016) reported consistent results in yogurts enriched with aronia juice, supporting the sensory acceptability of aronia when properly formulated. In contrast, a study by Kim et al. (2019) reported that the addition of aronia powder to milk, yogurt, and kefir led to a decrease in sensory scores as the concentration of the powder increased. In conclusion, the addition of aronia and probiotic supplementation prior to fermentation positively influenced the sensory quality of the yogurts in this study.

## 4 Conclusions

This study demonstrated that the addition of aronia puree (2% and 6%) and *L. acidophilus* DSM 20079, particularly when applied prior to fermentation, significantly enhanced the TPC, antioxidant capacity, and α‐glucosidase inhibitory activity of yogurt samples. During simulated gastrointestinal digestion, FRAP values increased across all yogurt formulations, suggesting improved ferric reducing capacity under the in vitro digestion conditions evaluated. While aronia addition improved WHC and reduced syneresis, it also led to a decrease in yogurt hardness. Moreover, higher aronia concentrations positively influenced visual appearance, whereas lower concentrations (2%) resulted in reduced color stability during storage. Although probiotic supplementation enhanced ACE inhibitory activity, the presence of aronia may have attenuated this effect, possibly due to interactions between polyphenols and milk proteins. This study was limited by the lack of phenolic profiling of the aronia puree and the use of an in vitro gastrointestinal digestion model. Future studies incorporating detailed phenolic characterization and in vivo validation are needed to further substantiate these findings.

## Author Contributions

**Aysun Oraç:** formal analysis, investigation, writing – review and editing. **Çiğdem Konak Göktepe:** conceptualization, methodology, software, validation, formal analysis, investigation, data curation, writing – original draft, writing – review and editing, visualization. **Neşe Mutlu:** methodology, formal analysis, investigation, writing – original draft, writing – review and editing. **Nihat Akin:** conceptualization, validation, resources, data curation, supervision, project administration, funding acquisition. **Lütfi Pirlak:** investigation, writing – review and editing. **Talha Demirci:** conceptualization, methodology, formal analysis, investigation, writing – original draft, writing – review and editing.

## Funding

This work was financially supported by the Scientific Research Projects Coordination Unit of Selcuk University (Grant No. 23401065).

## Ethics Statement

This study does not involve clinical human or animal experimentation. Sensory evaluation was conducted with trained adult panelists under non‐invasive conditions, and participation was voluntary.

## Conflicts of Interest

The authors declare no conflicts of interest.

## Data Availability

The datasets generated during and/or analyzed during this study are available from the corresponding author upon reasonable request.
